# Complete Genome Sequence and Genomic Characterization of *Lactobacillus acidophilus* LA1 (11869BP)

**DOI:** 10.3389/fphar.2018.00083

**Published:** 2018-02-08

**Authors:** Won-Hyong Chung, Jisu Kang, Mi Young Lim, Tae-joong Lim, Sanghyun Lim, Seong Woon Roh, Young-Do Nam

**Affiliations:** ^1^Research Group of Gut Microbiome, Korea Food Research Institute, Wanju, South Korea; ^2^Department of Food Biotechnology, Korea University of Science and Technology, Daejeon, South Korea; ^3^Research and Development Center, Cell Biotech Co., Ltd., Gimpo, South Korea; ^4^Microbiology and Functionality Research Group, World Institute of Kimchi, Gwangju, South Korea

**Keywords:** probiotics, *Lactobacillus acidophilus*, *Salmonella* infection, genome sequence, PacBio

## Introduction

Our body has natural defense systems to protect against potentially harmful microbes, including the physical and chemical barriers of the intestinal epithelium (Corfield et al., 2000). The physical barrier of the intestinal epithelium protects the host against pathogenic microbes (Anderson et al., 1993), and the intestinal mucosa coated with mucus excretes pathogens from the intestinal tract (Corfield et al., 2000).

The gut microbiota also controls the number of enteric pathogens by producing anti-microbial molecules, such as proteinaceous bacteriocins (Kamada et al., 2013), and by inhibiting the proliferation of pathogens by generating organic acids-short chain fatty acids (SCFAs) that lower the local pH (Shin et al., 2002), and by regulating the expression of virulence genes in certain pathogenic bacteria, such as *Salmonella* (Gantois et al., 2006). Moreover, the gut microbiota indirectly prevents pathogenic infection by enhancing the functions of the host mucosal barrier and innate immune system (Kobayashi et al., 2005). Therefore, in a healthy gastrointestinal tract, host cells and the gut microbiota synergistically protect the host from pathogenic infections.

The human intestinal tract harbors a dense population of resident microbiota, consisting of bacteria, archaea, viruses, and fungi. Host genetics, diet, and environmental insults can affect the gut microbial composition (Human Microbiome Project Consortium, 2012), and disruption of the gut microbiota can lead to invasion and overgrowth of certain pathogenic bacteria, such as virulent *Escherichia coli, Salmonella enterica* serovar *Typhimurium*, and *Clostridium difficile* (Bohnhoff et al., 1954; Rupnik et al., 2009; Ayres et al., 2012). In particular, the use of antibiotics can lead to a temporary or long-term reduction of bio-diversity, and this change in the gut microbiota, called dysbiosis, increases susceptibility to microbial infection and the proliferation of antibiotic-resistant strains (Vangay et al., 2015).

On the other hand, probiotics have been also considered for the treatment or prevention of diverse infectious diseases, and previous studies have reported the successful treatment of infection by pathogenic bacteria, including *Escherichia, Klebsiella, Shigella, Enterobacter, Pseudomonas, Clostridium, Helicobacter*, etc., (Kabir et al., 1997; Brashears et al., 1998; Forestier et al., 2001; Ogawa et al., 2001). Therefore, the use of probiotics could be an alternative strategy for the treatment or prevention of infectious disease that avoids the gut microbiota dysbiosis associated with antibiotic treatment.

In our previous study, we reported that *Lactobacillus acidophilus* strain LA1 (11869BP), an isolate from a dairy product, had preventive effects against *Salmonella* infection (Kim et al., 2013). While the anti-pathogenic effects of various probiotics have been widely demonstrated, the exact mechanism of action is not well understood. In addition, our genomic knowledge of *L. acidophilus* strains is insufficient because only five complete *L. acidophilus* genomes including LA1 are currently available in NCBI database (https://www.ncbi.nlm.nih.gov/genome/). Therefore, to gain better insight into its probiotic and *Salmonella* infection-preventing effects, we sequenced and analyzed the genome of *L. acidophilus* LA1. The availability of this genomic information will allow for further in-depth analysis and a better understanding of the probiotic functions of *L. acidophilus* strains for the prevention of pathogenic infections.

## Materials and methods

### Bacterial growth, DNA extraction, and sequencing

In our previous study, we isolated *L. acidophilus* LA1 from a fermented dairy product in Korea (Kim et al., 2013). To analyze the genomic content of strain LA1, we cultivated the bacteria in MRS medium (Difco, USA) at 37°C for 18 h. Genomic DNA was extracted and purified using the QIAamp DNA Mini Kit (Qiagen, Germany). The extracted genomic DNA was quantified with a NanoDrop 2000 UV-Vis spectrophotometer (Thermo Scientific, USA) and Qubit 2.0 fluorometer (Life Technologies, USA). The genome of *L. acidophilus* LA1 was sequenced with the PacBio RS II (Menlo Park, USA) sequencing platform.

### Gene annotation

Genome assembly was performed using HGAP 3.0 (Chin et al., 2013), with default options. The start position of the chromosome was determined by the location of the gene encoding the chromosomal replication initiation protein, *dnaA*, as well as the GC skew pattern. Annotation of this genome was carried out with the NCBI Prokaryotic Genome Annotation Pipeline (PGAAP) (Tatusova et al., 2016). From the ASN.1-formatted annotation file, the protein-coding genes and rRNA genes were extracted using the NCBI toolbox (http://www.ncbi.nlm.nih.gov/IEB/ToolBox). Coding genes were assigned to COG categories using BLASTP and the COG database (Galperin et al., 2015), with an *e*-value cutoff of 1e-3.

### Pan-genome comparison

Nine complete genome sequences that belong to *L. acidophilus* group were selected for phylogenetic and comparative analysis: *L. acidophilus* NCFM (assembly accession: GCF_000011985.1), *L. acidophilus* La-14 (GCF_000389675.2), *L. acidophilus* FSI4 (GCF_000934625.1), *L. gallinarum* HFD4 (GCF_001314245.2), *L. helveticus* CNRZ32 (GCF_000422165.1), *L. crispatus* ST1 (GCF_000091765.1), *L. kefiranofaciens* ZW3 (GCF_000214785.1), *L. amylovorus* GRL1118 (GCF_000194115.1), and *L. acetotolerans* NBRC 13120 (GCF_001042405.1). *L. acetotolerans* NBRC 13120 was used as the out-group in the phylogenetic analysis. The three *L. acidophilus* genomes (NCFM, La-14, and FSI4) and the present sequenced genome, *L. acidophilus* LA1, were used for the comparative analysis.

A phylogenetic tree based on the 16S gene sequences was constructed by the maximum likelihood method based on the Tamura-Nei model (Tamura and Nei, 1993). Orthologous average nucleotide identity (OrthoANI) between genome sequences was computed (Lee et al., 2016). To obtain genomic distance, the OrthoANI values were converted to distance values with the following formula: distance = 1 – (OrthoANI/100). Evolutionary distance was computed from the genome-distance matrix using the neighbor-joining method (Saitou and Nei, 1987). The tree is drawn to scale, with branch lengths presented in the same units as those for the evolutionary distances used to infer the phylogenetic tree. The phylogenetic tree was generated using MEGA6 (Tamura et al., 2013).

A pan-genomic study was performed to investigate the functional conservation in the sequenced genome by using GET_HOMOLOGOUS (Contreras-Moreira and Vinuesa, 2013). COG triangles was used as the clustering method, and a minimum of 50% amino acid identity and 50% coverage were used for the clustering threshold.

Carbohydrate active enzymes (CAZymes) were searched by using the CAZymes analysis tool kit (Park et al., 2010; Lombard et al., 2014). Different CAZy families were used to identify the key enzymes related to polysaccharide degradation: glycoside hydrolases (GHs), glycosyl transferases (GTs), carbohydrate esterases (CEs), auxiliary activities (AAs), and carbohydrate-binding modules (CBMs).

Bacteriocins were predicted with BAGEL 3 which is a web service for bacteriocin searches that uses a bacteriocin mining tool (Van Heel et al., 2013). BAGEL 3 is based on three types of databases: modified bacteriocins, unmodified bacteriocins, and post-translationally modified peptides.

### Unique genomic features

The CRISPR regions were identified with a CRISPR on-line detection tool, CRISPR finder (Grissa et al., 2007). The sequences of dicers and spacers were downloaded from the analysis server after the finding computation. Aligned pairs of spacers in *L. acidophilus* NCFM and LA1 were obtained by running BLASTN without the dust masking option. The numerical order of spacers in LA1 followed the order in NCFM. To find counterparts for the spacers, a BLASTN search was performed against the NCBI NT and NR database (downloaded on 16 May, 2017), with no dust option and an *e*-value of 0.1. The search result was filtered with 90% identity and 80% coverage.

Prophage insert regions were detected with an on-line phage search tool, PHASTER (Arndt et al., 2016). The genomic structure of the inserted prophage and the associated genes were obtained from the computation result. The detected ORFs were annotated based on the highest hit in a BLAST search against the annotated bacterial genome database in the PHASTER system.

## Results and discussion

### Genome features

#### Overall features of the LA1 genome

**Figure 1:**
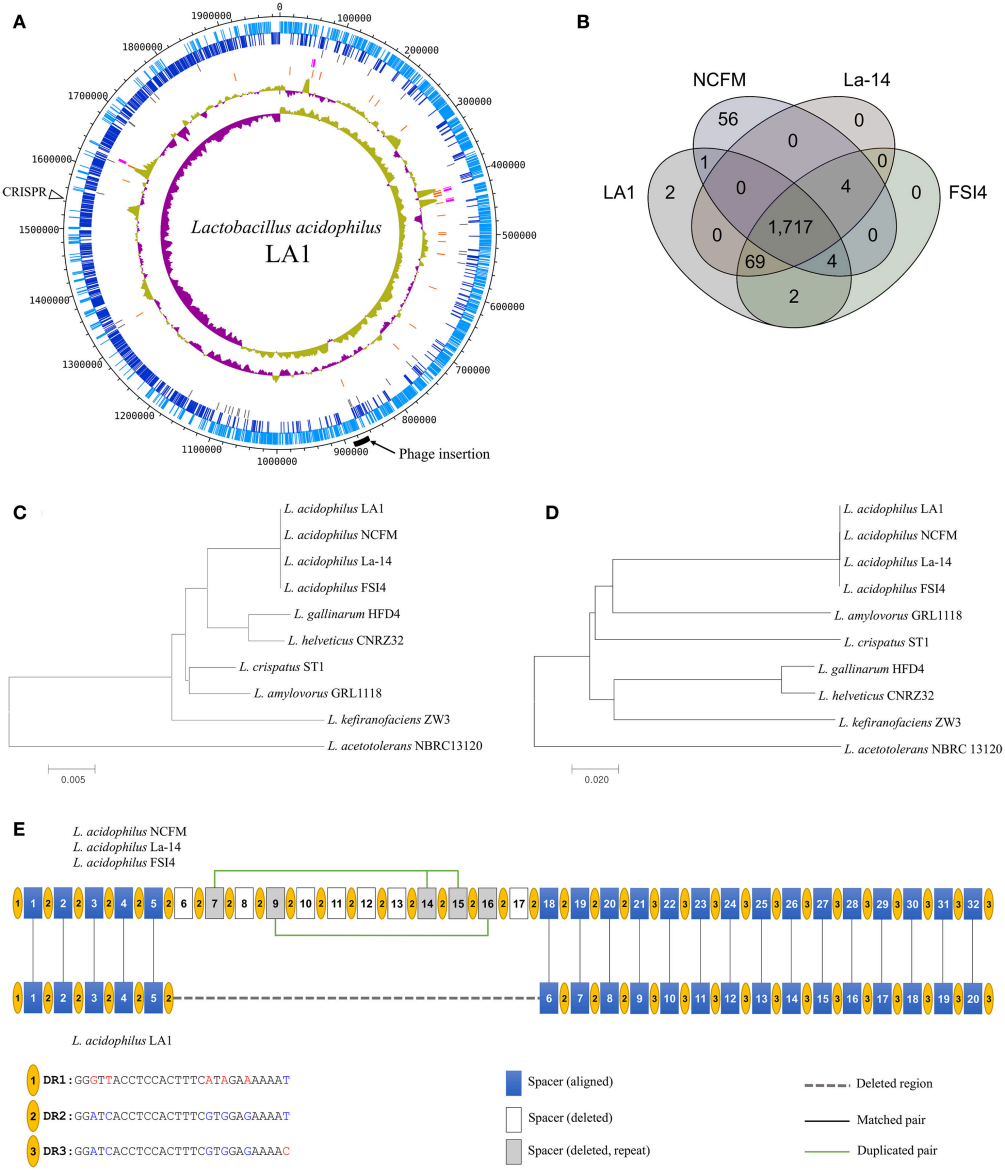
**(A)** Circular map of the *Lactobacillus acidophilus* LA1 genome. Tracks (from outset): forward-strand coding CDS, reverse-strand coding CDS, pseudogenes, rRNAs, tRNAs, G+C content, and GC skew. **(B)** Venn diagram of the shared gene clusters among the four *L. acidophilus* genomes. **(C)** Phylogenetic tree of the *L. acidophilus* groups based on 16S rRNA genes and **(D)** phylogenetic tree based on genome-wide identities (ANI). **(E)** Comparison of the CRISPR structure in LA1 and the other *L. acidophilus* genomes. Yellow circles indicate the dicers. The blue, white, and gray boxes represent the spacers that are aligned in LA1, the spacers deleted in LA1, and the deleted repeat spacers in LA1, respectively. A dashed line fills the deleted region in LA1. Vertical lines indicate aligned pairs of spacers. Green lines link the repeat spacers.

We obtained the complete genome sequence of *L. acidophilus* LA1 using SMRT sequencing, which showed that the genome is composed of a 1.99-Mbp circular chromosome with 34.7% G+C content (Table 1). A total of 1,953 genes were identified in the LA1 genome, including 1,844 protein-coding genes, 76 RNA genes, and 33 pseudo genes. Four sets of ribosomal RNA genes, including 5S, 16S, and 23S genes, were also found. Other RNA genes, including 61 tRNA genes and three non-coding RNA (or ncRNA) genes were found. We also found ncRNA-coding sequences in *L. acidophilus* NCFM, even though they were not reported in the genome annotation. We hypothesized that these ncRNAs were absent from the annotation because of the difference in the annotation methods, since these types of ncRNA genes were recently introduced in the annotation system (PGAAP 3.1 and above). These genomic features of LA1 are shown in Figure 1A.

**Table 1:** Genome features of *Lactobacillus acidophilus* LA1.

**Attribute**	**Values**
Accession number	CP017062
Genome size (bp)	1,991,195
No. of sequences	1 chromosome (0 plasmid)
Assembly status	Complete
Genes	1,953
Coding genes	1,844
RNA genes	76
rRNAs (5S, 16S, 23S)	12 (4, 4, 4)
tRNAs	61
ncRNAs	3
Pseudo genes	33
CRISPRs	1
Phage insertion	1

#### Functional classification of LA1

Functional classifications of the protein-coding genes of LA1 were categorized into 26 COG functional codes (Supplementary Figure S1, Supplementary Table S2). Of the 1,844 protein-coding genes in LA1, 1,553 were assigned to COG, and 291 genes were not assigned. Because 135 genes were assigned multiple codes, the number of COG codes was 1,705. The percentage of proteins with unknown function, including “General function prediction only (R),” “Function unknown (S),” and “Not assigned (–),” was 29.3%. The four analyzed *L. acidophilus* strains, including LA1, have nearly identical numbers of functional genes, and they showed >99% genomic identity (Supplementary Table S3).

### Comparative analysis of the *L. acidophilus* group

#### Phylogenetic comparison

*Lactobacillus acidophilus* strains including LA1 showed high conservation each other by comparison of the *L. acidophilus* group (Bull et al., 2013, 2014), a subgroup of *Lactobacillus*. A comparative study of the 10 complete genomes in the *L. acidophilus* group was performed to confirm the genomic distance based on the 16S rRNA genes and average nucleotide identity (ANI). The genomic similarities ranged from 75 to 99.9%, and the similarities based on the 16S rRNA genes ranged from 94 to 100% (Supplementary Table S3). Four *L. acidophilus* strains were located on the same node of the both 16S rRNA phylogenetic and ANI trees (Figures 1C,D). Those genomes had identical 16S rRNA sequences and very similar ANI values (≥99%); therefore, the *L. acidophilus* genomes were difficult to distinguish by sequence similarity. We found different phylogenetic relationships between the 16S rRNA sequence-based and ANI value-based phylogenetic trees. *L. gallinarum* and *L. helveticus* were the closest to *L. acidophilus* on the 16S rRNA phylogenetic tree (Figure 1C). However, *L. amylovorus* was the closet to *L. acidophilus* on the ANI phylogenetic tree (Figure 1D). Phylogenetic studies using ANI better reflect the functional relationship between strains than studies based on 16S rRNA sequences, as shown by the statistics for COG categories. *L. gallinarum* and *L. helveticus* (marked as triangles in Supplementary Figure S1) showed distinguishable profiles when compared to the *L. acidophilus* genomes, and there was a greater than five-fold difference in “Mobilome: prophages, transposons (X).”

#### Pan-genome analysis

To obtain better insight into the specific features of LA1, we compared LA1 to nine complete genomes in the *L. acidophilus* group (Supplementary Table S1). A distinguishing feature of the four *L. acidophilus* genomes (LA1, NCFM, La-14, and FSI4) was the lower G+C content (2–3%) compared to the other species. Gene clustering was performed to determine the differences between these 10 genomes based on COG functional annotation (Supplementary Table S4). The genes were categorized into 3,810 functional clusters using the pan-genome analysis program, GET_HOMOLOGOUS. The four *L. acidophilus* genomes were compared by orthologous protein clustering to determine the LA1-specific genes (Figure 1B). Among the total 1,955 gene clusters, we found 1,717 core gene clusters. Only two gene clusters were identified as LA1 strain specific. Even though there were high similarities between the *L. acidophilus* genomes, four gene clusters were absent in the LA1 genome. Among the other *L. acidophilus* strains, 125 gene clusters were unique; 56 clusters belonged to NCFM, and the 69 clusters belonged to the other strains.

#### Carbohydrate-active enzymes (CAZymes)

Carbohydrate active enzymes (CAZymes) analysis showed that LA1 contains 344 genes in the five CAZymes gene families (Supplementary Table S5); 148 glycoside hydrolase (GH) genes, 130 glycosyl transferase (GT) genes, 25 carbohydrate esterase (CE) genes, 11 auxiliary activity (AA) genes, and 30 CBMs. These numbers of carbohydrate-active enzymes were relatively larger than those in *Lactobacillus plantarum*, which is an important probiotic species. For example, *L. plantarum* KLDS1.0391 contains only 34 GHs, 23 GTs, 14 CEs, 2 AAs, and 21 CPMs (Jia et al., 2017). GTs that catalyze the transfer of sugars from activated donor molecules to specific acceptors are important for the formation of surface structures recognized by host immune systems (Mazmanian et al., 2008). Therefore, the six-fold larger number of GT genes in *L. acidophilus* LA1 compared to *L. plantarum* KLDS1.0391 suggests the probiotic potential of LA1, especially for immune stimulation and pathogen defense.

#### Bacteriocin-related genes

*Lactobacillus acidophilus* genomes have three area(s) of interest (AOI) that include one class II bacteriocin and two class III bacteriocins in common: AOI_1 (bacteriocin III, Enterolysin A), AOI_2 (bacteriocin III, Helventicin J), and AOI_3 (bacteriocin II, Acidocin J) (Supplementary Table S6). Enterolysin A is a cell wall-degrading bacteriocin (Nilsen et al., 2003) with broad-spectrum antibiotic activity that acts by cleaving stem peptide bonds and lysing peptidoglycan in cell walls (Riley and Chavan, 2007; Khan et al., 2013). Helveticin J is antimicrobial protein that was first characterized in *L. helveticus* 481 (Joerger and Klaenhammer, 1986). However, a recent report revealed that helveticin is also found in other Lactobacillus species (Collins et al., 2017). Acidocin J is a heat-stable class II bacteriocin, which is mainly found in *L. acidophilus* (Riley and Chavan, 2007; Yang et al., 2014). In this study, Enterolysin A and Helventicin J were found in all *Lactobacillus* spp. whereas Acidocin J was only encoded in *L. acidophilus* genomes. In addition, all *L. acidophilus* genomes, except for *L. acidophilus* NCFM, have the same start position for each AOI.

### Unique characteristics of the LA1 genome

#### CRISPR region

We found a large deletion in the CRISPR region in LA1 when compared to NCFM, La-14, and FSI4. All the genomes, except LA1, have the same structure in the CRISPR region, with three types of 28-bp dicer sequences and 32 spacer sequences, whereas LA1 has only 20 spacer sequences (Figure 1E). The clean-cut of 12 spacers (6–17) was observed in this region. The CRISPR region has three types of dicers, and the deletion in LA1 was located in the middle of the array of type 2 dicers (DR2). The deleted spacers in LA1 include five duplicated spacers of two types, one type consists of spacers 7, 14, and 15, and the other consists of spacers 9 and 16. Considering the high conservation of the CRISPR region, this deletion may be a very recent event. Because one of the major roles of the CRISPR region is defense against foreign DNA (Yin et al., 2013), this shortage of spacers to detect unwanted nucleic acids in LA1 may lower its immune power. The structural variation in the CRISPR region can be found even within the same species, and could be used to interpret the evolutional history of a strain because the CRISPR region may reflect the phages that have tried to invade the cell. Therefore, the CRISPR region has been recently proposed for the identification of industrially important microorganisms (Barrangou and Horvath, 2012). Therefore, this difference in the CRISPR region can be used as a marker for distinguishing LA1 from other *L. acidophilus* strains.

#### Prophage insertion

A 26-kbp prophage insert was identified in the LA1 genome at chromosomal position 863,940–890,001 (Supplementary Table S7). The position and size of the insert region were similar to that in other *L. acidophilus* genomes, except for the number of proteins encoded in that region. Despite their high similarity, the number of genes varies from 10 to 15 (Supplementary Table S8). LA1 contains the smallest number of genes, FSI4 has the largest number of genes, and NCFM and La-14 have the same number of genes (12). Genes required for phage invasion, including *attL* (ORF1), phage integrase (ORF6), and *attR* (ORF15), were found in the inserted region. LA1 showed the highest rate of decay in the inserted phage genome, and five genes, ORF2–5 and ORF9, were degraded when compared to FSI4.

## Conclusion

Here, we sequenced and analyzed the complete genome of a probiotic strain with the potential to prevent *Salmonella* infection, *L. acidophilus* LA1. In the current study, we demonstrated that the LA1 genome contains the genes required for the biosynthesis of the three bacteriocins, Enterolysin A, Helveticin J, and Acidocin J. In addition, the six-fold larger number of GT genes in LA1 compared to the number in one of the most well-described probiotic strains, *L. plantarum* KLDS1.0391, suggests the probiotic potential of LA1, especially in terms of immune stimulation and fortification of pathogen defense. Interestingly, one of the most unique features of LA1 when compared to the other currently available *L. acidophilus* genome sequences is the large, clean-cut of 12 spacers from the CRISPR region and the difference in the historical record of phage infection, which can be used as a genetic marker for identification of this industrially and medically important probiotic strain to distinguish it from genetically related *L. acidophilus* strains. Considering the possible use of *L. acidophilus* LA1 as beneficial probiotic, the availability of the LA1 genome is an important step for understanding its evolution and probiotic function against pathogenic bacteria.

## Data access

The *Lactobacillus acidophilus* LA1 genome sequencing project has been deposited into GenBank under accession number CP017062. This strain has been deposited in the Korean Collection for Type Cultures (deposit ID: KCTC 11906BP).

## Author contributions

Y-DN and SR: Designed and coordinated all the experiments; T-jL and SL: Performed the bacterial cultivation, and DNA extraction and purification; W-HC, JK, and ML: Performed the genome analysis; W-HC, JK, and Y-DN: prepared the manuscript; All authors have read and approved the manuscript.

### Conflict of interest statement

The authors declare that the research was conducted in the absence of any commercial or financial relationships that could be construed as a potential conflict of interest.
