# Supplementary material for: Complete Genome Sequence and Genomic Characterization of Lactobacillus acidophilus LA1 (11869BP)
Source: Front Pharmacol. 2018 Feb 8;9:83. doi: 10.3389/fphar.2018.00083 (PMC5809850; doi:10.3389/fphar.2018.00083)
Supplement: Supplementary file 1 [file DataSheet1.pdf]

Supplementary materials for "Complete genome sequence and genomic characterization of *Lactobacillus acidophilus* LA1 (11869BP)"

Supplementary fig. S1. Classification of genes by COG.

Supplementary table S1. Summary of the complete genomes in *Lactobacillus acidophilus* group

Supplementary table S2. Classification of genes by COG codes

Supplementary table S3. Similarity values between genomes

Supplementary table S4. Top 20 clusters of pan-genomic clustering result of *Lactobacillus acidophilus* group

Supplementary table S5. Number of genes associated with five CAZymes gene families

Supplementary table S6. Summary of bacteriocin in the genomes of *L. acidophilus* group

Supplementary table S7. Phage sequences found in the *L. acidophilus* genomes

Supplementary table S8. Detailed structure of the prophage ORFs inserted to the *L. acidophilus* genomes

Supplementary fig. S1. Classification of genes by COG. *L. acidophilus* strains LA1, NCFM, La-14, and FSI4 are marked with circles. *L. gallinarum* and *L. helveticus* are marked with triangles. All other genomes are marked with diamonds.

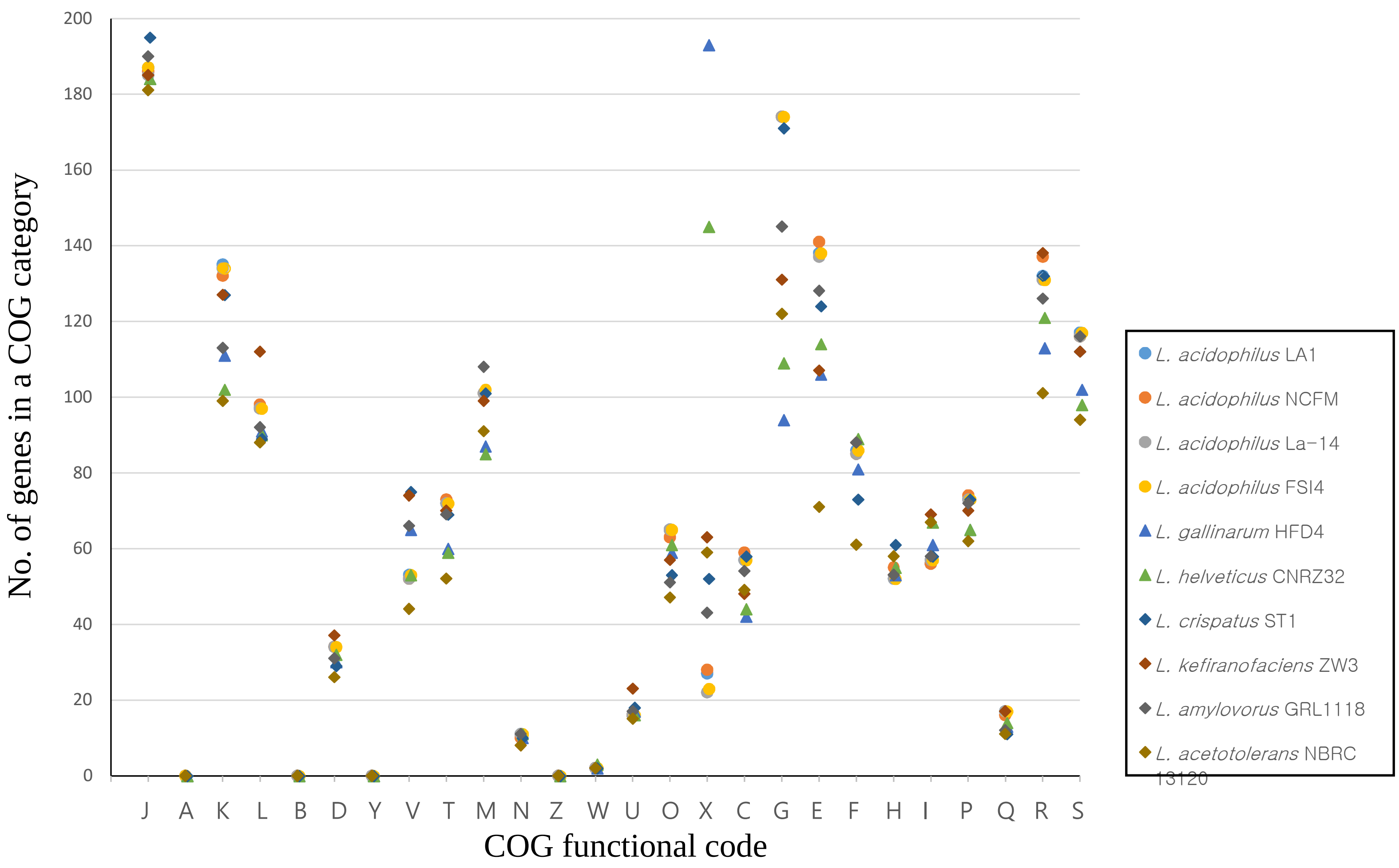

Supplementary table S1. Summary of the complete genomes in *Lactobacillus acidophilus* group.

Genome						Annotation							
Full name	Accession	Sequence	Total size (bp)	GC (%)	Assembly level	Genes	Coding	RNAs	rRNAs (5S,16S,23S)	tRNAs	ncRNAs	Pseudo	CRISPRs
L. acidophilus LA1	CP017062	1 chrom	1,991,195	34.7	Complete	1,953	1,844	76	4, 4, 4	61	3	33	1
L. acidophilus NCFM	NC_006814	1 chrom	1,993,560	34.7	Complete	1,927	1,832	74	4, 4, 5	61	0	0	1
L. acidophilus La-14	NC_021181	1 chrom	1,991,579	34.7	Complete	1,948	1,835	73	4, 4, 4	61	0	40	1
L. acidophilus FSI4	NZ_CP010432	1 chrom	1,991,969	34.7	Complete	1,948	1,845	73	4, 4, 4	61	0	30	1
L. gallinarum HFD4	NZ_CP012890, NZ_CP012891, NZ_CP012892, NZ_CP012893, NZ_CP012895, NZ_CP012896	1 chrom, 5 plasmids	2,374,782	36.8	Complete	2,410	1,950	82	5, 5, 5	64	3	378	1
L. helveticus CNRZ32	NC_021744	1 chrom	2,225,962	36.9	Complete	2,273	1,913	75	4, 4, 4	63	0	285	0
L. crispatus ST1	NC_014106	1 chrom	2,043,161	36.9	Complete	2,011	1,840	76	4, 4, 4	64	0	95	3
L. kefiranofaciens ZW3	NC_015602	1 chrom	2,113,023	37.7	Complete	2,067	1,787	72	4, 4, 4	60	0	208	1
L. amylovorus GRL1118	NC_017470, NC_017471, NC_017472	1 chrom, 2 plasmids	1,977,087	38.0	Complete	1,975	1,815	74	4, 4, 4	62	0	86	3
L. Acetotolerans NBRC_13120	NZ_AP014808	1 chrom	1,704,859	36.7	Complete	1,669	1,492	75	4, 4, 4	60	3	102	0

Supplementary table S2. Classification of genes by COG codes. CODE indicates 26 COG functional categories and a not assigned ('-') category. The description of each code was placed the right-side of the table. The genome names were abbreviated to their strain names.

CODE	LA1	NCFM	La-14	FSI4	HFD4	CNRZ32	ST1	ZW3	GRL1118	NBRC 13120	Description
J	187	186	185	187	184	184	195	185	190	181	Translation, ribosomal structure and biogenesis
A	0	0	0	0	0	0	0	0	0	0	RNA processing and modification
K	135	132	134	134	111	102	127	127	113	99	Transcription
L	97	98	97	97	91	90	89	112	92	88	Replication, recombination and repair
B	0	0	0	0	0	0	0	0	0	0	Chromatin structure and dynamics
D	34	34	34	34	30	32	29	37	31	26	Cell cycle control, cell division, chromosome partitioning
Y	0	0	0	0	0	0	0	0	0	0	Nuclear structure
V	53	52	52	53	65	53	75	74	66	44	Defense mechanisms
T	72	73	72	72	60	59	69	70	69	52	Signal transduction mechanisms
M	101	101	101	102	87	85	101	99	108	91	Cell wall/membrane/envelope biogenesis
N	11	10	11	11	10	11	10	8	11	8	Cell motility
Z	0	0	0	0	0	0	0	0	0	0	Cytoskeleton
W	2	2	2	2	2	3	2	2	2	2	Extracellular structures
U	16	16	16	16	17	16	18	23	17	15	Intracellular trafficking, secretion, and vesicular transport
O	65	63	65	65	59	61	53	57	51	47	Posttranslational modification, protein turnover, chaperones
X	27	28	22	23	193	145	52	63	43	59	Mobilome: prophages, transposons
C	57	59	57	57	42	44	58	48	54	49	Energy production and conversion
G	174	174	174	174	94	109	171	131	145	122	Carbohydrate transport and metabolism
E	138	141	137	138	106	114	124	107	128	71	Amino acid transport and metabolism
F	86	85	85	86	81	89	73	88	88	61	Nucleotide transport and metabolism
H	52	55	52	52	53	55	61	53	53	58	Coenzyme transport and metabolism
I	56	56	57	57	61	67	58	69	58	67	Lipid transport and metabolism
P	73	74	73	73	65	65	73	70	72	62	Inorganic ion transport and metabolism
Q	17	16	17	17	13	14	11	17	12	11	Secondary metabolites biosynthesis, transport and catabolism
R	132	137	131	131	113	121	132	138	126	101	General function prediction only
S	117	116	116	117	102	98	112	112	116	94	Function unknown
-	291	278	292	292	332	332	286	423	304	171	Not assigned

Supplementary table S3. Similarity values between genomes. The values in lower right triangle indicate pairwise similarities of average nucleotide identities (ANIs) and the values in upper left triangle indicate pairwise similarity of 16S rRNA genes.

	LA1	NCFM	La-14	FSI4	HFD4	CNRZ32	ST1	ZW3	GRL1118	NBRC_13120
L. acidophilus LA1		100	100	100	98.3450	98.4087	98.4723	97.2629	98.3450	94.0166
L. acidophilus NCFM	99.9894		100	100	98.3450	98.4087	98.4723	97.2629	98.3450	94.0166
L. acidophilus La-14	99.9924	99.9855		100	98.3450	98.4087	98.4723	97.2629	98.3450	94.0166
L. acidophilus FSI4	99.9951	99.9854	99.9921		98.3450	98.4087	98.4723	97.2629	98.3450	94.0166
L. gallinarum HFD4	80.7025	80.7737	80.6827	80.6082		99.1725	98.2813	97.0083	98.0267	94.3348
L. helveticus CNRZ32	80.6914	80.6734	80.5434	80.5867	97.2932		98.4723	97.0719	98.0904	94.2712
L. crispatus ST1	79.9150	79.8429	79.9097	79.8981	80.2848	80.2904		97.8358	98.8542	94.3348
L. kefiranofaciens ZW3	79.2413	79.2668	79.2930	79.3827	82.7279	82.6864	79.7087		97.5812	93.5710
L. amylovorus GRL1118	81.7273	81.7232	81.8051	81.7506	81.2886	81.5137	80.2846	79.9894		94.3985
L. acetotolerans NBRC_13120	74.9473	74.9667	74.9196	75.1009	75.6046	75.4919	74.9250	75.4821	75.0343	

Supplementary table S4. Top 20 clusters of pan-genomic clustering result of *Lactobacillus acidophilus* group. Cluster name is derived from the description of gene clusters which were generated by get_homologues. Cluster size is the sum of genes associated with the cluster. This table shows the top 20 clusters among the LA1 gene clusters.

**Cluster name**	**Cluster size**	**LA1**	**NCFM**	**La-14**	**FSI4**	**HFD4**	**CNRZ32**	**ST1**	**ZW3**	**GRL1118**	**NBRC 13120**
5586_integrase	45	6	6	5	6	2	4	0	4	1	11
6517_acetate_kinase	30	4	4	4	4	2	2	3	3	3	1
360_transposase	22	4	3	3	4	0	0	1	2	5	0
5832_ATP-dependent_Clp_pr..	30	3	3	3	3	3	3	3	3	3	3
7004_guanine_permease	25	3	2	2	3	1	3	3	3	3	2
6723_calcium-translocatin..	22	3	3	3	3	2	1	3	1	2	1
6133_peptide_ABC_transpor..	22	3	3	3	3	2	3	2	0	2	1
6888_sugar_ABC_transporte..	20	3	3	3	3	0	1	3	1	2	1
957_mucus-binding_protei..	12	3	3	3	3	0	0	0	0	0	0
73_transposase	27	2	2	1	2	2	12	4	0	2	0
5529_gyrB	20	2	2	2	2	2	2	2	2	2	2
5690_peptidase_M13	20	2	2	2	2	3	3	2	1	2	1
5727_aminopeptidase	19	2	2	2	2	2	2	2	2	2	1
5973_glycerol_phosphate_l..	18	2	2	2	2	1	1	2	2	2	2
116_transcriptional_regu..	17	2	2	2	2	2	1	3	1	2	0
6327_formate--tetrahydrof..	17	2	2	2	2	2	2	0	2	2	1
181_peptide_ABC_transpor..	16	2	2	2	2	0	2	2	2	2	0
6686_MFS_transporter	16	2	2	2	2	1	1	2	1	2	1
6620_oleate_hydratase	16	2	2	2	2	0	1	2	2	2	1
803_aspartate_kinase	16	2	2	2	2	2	1	2	2	1	0

Supplementary table S5. Number of genes associated with five CAZymes gene families.

	LA1	NCFM	La-14	FSI4	HFD4	CNRZ32	ST1	ZW3	GRL1118	NBRC 13120
glycoside hydrolases (GHs)	148	149	143	144	139	151	152	128	129	109
glycosyl transferases (GTs)	130	129	130	130	106	111	127	118	136	98
carbohydrate esterases (CEs)	25	25	25	25	27	29	26	34	27	24
auxiliary activities (AAs)	11	12	11	11	13	9	12	15	10	11
carbohydrate-binding modules (CBMs)	30	28	30	30	22	22	29	24	27	24

Supplementary Table S6. Summary of bacteriocin in the genomes of *L. acidophilus* group.

**Strain**	**Area Of Interest**	**Class**	**Identified on the basis of**	**BAGEL3 prediction**	**AOI start position**	**Relative position**
L. acidophilus LA1	AOI_1	>10kd	Blast_bacteriocinIII	Enterolysin A	1160238	10000-10665
	AOI_2	>10kd	Blast_bacteriocinIII	Helveticin J	1548041	10000-10977
	AOI_3	Unmodified	Blast_bacteriocinII	Acidocin J	1806351	10000-10251
L. acidophilus NCFM	AOI_1	>10kd	Blast_bacteriocinIII	Enterolysin A	1160302	10000-10665
	AOI_2	>10kd	Blast_bacteriocinIII	Helveticin J	1548847	10000-10977
	AOI_3	Unmodified	Blast_bacteriocinII	Acidocin J	1803125	15587-15838
L. acidophilus La-14	AOI_1	>10kd	Blast_bacteriocinIII	Enterolysin A	1159879	10000-10665
	AOI_2	>10kd	Blast_bacteriocinIII	Helveticin J	1548422	10000-10977
	AOI_3	Unmodified	Blast_bacteriocinII	Acidocin J	1806732	10000-10251
L. acidophilus FSI4	AOI_1	>10kd	Blast_bacteriocinIII	Enterolysin A	1160590	10000-10665
	AOI_2	>10kd	Blast_bacteriocinIII	Helveticin J	1549126	10000-10977
	AOI_3	Unmodified	Blast_bacteriocinII	Acidocin J	1807429	10000-10251
L. gallinarum HFD4	AOI_1	>10kd	Blast_bacteriocinIII	Helveticin J	282412	10000-10965
	AOI_2	>10kd	Blast_bacteriocinIII	Helveticin J<br>Enterolysin A	1831721	17354-18301<br>10000-10656
	AOI_3	>10kd	Blast_bacteriocinIII	Enterolysin A	1856564	10000-10164
	AOI_4	>10kd	Blast_bacteriocinIII	Helveticin J	2276176	11815-12069<br>11501-11851<br>8299-8628<br>10000-10179
L. helveticus CNRZ32	AOI_1	>10kd	Blast_bacteriocinIII	Enterolysin A	1373925	10000-10656
	AOI_2	>10kd	Blast_bacteriocinIII	Helveticin J	1772120	10000-10977
	AOI_3	>10kd	Blast_bacteriocinIII	Helveticin J	2079607	10000-10965
L. crispatus ST1	AOI_1	>10kd	Blast_bacteriocinIII	Helveticin J	682	10000-10878
	AOI_2	>10kd	Blast_bacteriocinIII	Enterolysin A	1186707	10000-10674
	AOI_3	>10kd	Blast_bacteriocinIII	Enterolysin A	1218163	10000-10623
	AOI_4	>10kd	Blast_bacteriocinIII	Helveticin J	1534811	10000-10980
	AOI_5	Unmodified	Blast_bacteriocinII	Putative bacteriocin	1617600	10000-10638
L. kefiranofaciens ZW3	AOI_1	>10kd	Blast_bacteriocinIII	Helveticin J	237929	10000-10977
	AOI_2	>10kd	Blast_bacteriocinIII	Enterolysin A	504364	10000-10446
L. amylovorus GRL1118	AOI_1	>10kd	Blast_bacteriocinIII	Helveticin J	67942	14579-15517<br>10000-11028
	AOI_2	>10kd	Blast_bacteriocinIII	Helveticin J	362073	10000-11052
	AOI_3	>10kd	Blast_bacteriocinIII	Enterolysin A	1179529	10000-10668
	AOI_4	>10kd	Blast_bacteriocinIII	Helveticin J	1529453	10000-10977
L. acetotolerans NBRC_13120	AOI_1	>10kd	Blast_bacteriocinIII	Helveticin J	35784	10000-10980
	AOI_2	>10kd	Blast_bacteriocinIII	Enterolysin A	853868	10000-10644
	AOI_3	>10kd	Blast_bacteriocinIII	Helveticin J	1612611	10000-10959
	AOI_4	Unmodified	Blast_bacteriocinII	Pediocin	1660316	10000-10491

Supplementary table S7. Phage sequences found in the *L. acidophilus* species

Strain	Region Length	Completeness	Score	Total Proteins	Region Position	Most Common Phage	GC %
LA1	26Kb	incomplete	30	8	863940-890001	PHAGE_Lactob_Lj965_NC_005355(2)	33.41%
NCFM	26Kb	incomplete	20	9	863952-890013	PHAGE_Lactob_Lj965	33.41%
La-14	26Kb	incomplete	20	9	863948-890009	PHAGE_Lactob_prophage_Lj965_NC_005355(3)	33.41%
FSI4	26Kb	incomplete	30	12	864244-890305	PHAGE_Lactob_Lj965_NC_005355(3)	33.41%

Supplementary table S8. Detailed structure of the prophage ORFs associated to the *L. acidophilus* genomes. At most fifteen ORFs were detected from ORF1(*attL*) to ORF15(*attR*). "=" means the same annotation between genomes and "?" means the different annotation between genomes at each aligned ORF location.

	NCFM			La-14			FSI4			LA1	
	**CDS_POSITION**	**BLAST_HIT**		**CDS_POSITION**	**BLAST_HIT**		**CDS_POSITION**	**BLAST_HIT**		**CDS_POSITION**	**BLAST_HIT**
ORF1	863952..863969	attL	=	863948..863965	attL	=	864244..864261	attL	=	863940..863957	attL
ORF2							868045..868878	DegV domain-containing protein			
ORF3							c(868929..869504)	membrane protein			
ORF4	c(869317..870294)	hypothetical protein FR483_N093R	=	c(869313..870290)	hypothetical protein FR483_N093R	?	c(869609..870586)	choloylglycine hydrolase			
ORF5	870429..870517	tRNA	=	870425..870513	tRNA	=	870721..870809	tRNA			
ORF6	c(870710..871660)	phage integrase	=	c(870706..871656)	phage integrase	=	c(870971..872083)	phage integrase	=	c(870698..871648)	phage integrase
ORF7	c(871880..872080)	hypothetical protein	=	c(871876..872076)	hypothetical protein	=	c(872172..872372)	hypothetical protein	=	c(871868..872068)	hypothetical protein
ORF8							c(872375..873028)	tail tape measure protein	=	c(872071..872724)	tail tape measure protein
ORF9	c(872083..872736)	putative superinfection immunity protein	=	c(872079..872732)	putative superinfection immunity protein	?	c(873075..873548)	putative repressor			
ORF10	c(873265..873603)	putative repressor	=	c(873261..873599)	putative repressor	=	c(873557..873895)	putative repressor		c(873253..873591)	putative repressor
ORF11	873767..873970	putative cro repressor	=	873763..873966	putative cro repressor	=	874059..874262	putative cro repressor	=	873755..873958	putative cro repressor
ORF12	874230..875621	transporter protein	?	874226..875617	amino acid permease domain protein	=	874522..875913	amino acid permease	?	874218..875609	hypothetical protein
ORF13	875749..876705	putative thymidylate synthase	=	875745..876701	putative thymidylate synthase	=	876041..876997	thymidylate synthase	=	875737..876693	thymidylate synthase
ORF14	876715..877218	putative dihydrofolate reductase	?	876711..877214	gp125	=	877007..877510	gp125	=	876703..877206	gp125
ORF15	890013..890030	attR	=	890009..890026	attR	=	890305..890322	attR	=	890001..890018	attR
